# Extramedullary Disease—Achilles Heel in Myeloma?

**DOI:** 10.1002/ajh.70138

**Published:** 2025-12-29

**Authors:** Shaji Kumar, Joshua Richter, Saad Z. Usmani, Yael C. Cohen, Jing Christine Ye, María‐Victoria Mateos, Vania Hungria, Elena Zamagni

**Affiliations:** ^1^ Mayo Clinic Rochester Rochester Minnesota USA; ^2^ Mount Sinai School of Medicine New York New York USA; ^3^ Memorial Sloan Kettering Cancer Center New York New York USA; ^4^ Tel Aviv Sourasky (Ichilov) Medical Center Tel Aviv Israel; ^5^ Faculty of Medical & Health Sciences, Tel Aviv University Tel Aviv Israel; ^6^ MD Anderson Cancer Center, University of Texas Houston Texas USA; ^7^ Hospital of Salamanca/IBSAL/CIC/CIBERONC Salamanca Spain; ^8^ Clinica Médica São Germano São Paulo Brazil; ^9^ University of Bologna Bologna Italy

**Keywords:** bispecific antibodies, immunotherapy, paramedullary, relapsed myeloma

## Abstract

Despite advances in therapy, extramedullary disease (EMD) remains an aggressive form of multiple myeloma associated with poor outcomes. Patients with true EMD, in which plasmacytomas have become completely independent of bone, have a particularly poor prognosis. The pathogenesis of EMD is driven by complex mechanisms involving loss of adhesion molecules, heterogeneous genetic and epigenetic changes, and a solid tumor‐like architecture within the microenvironment. Although the introduction of advanced imaging techniques and immunotherapy has led to improved detection and more promising outcomes and regimens, respectively, more prospective studies dedicated to true EMD are needed.

## 1 Introduction

Multiple myeloma (MM) is a clonal plasma cell malignancy that originates in the terminally differentiated plasma cell [1]. It is characterized by plasma cells that accumulate in the bone marrow (BM), with varying proportions of the tumor cells in the peripheral blood depending on the stage of the disease. While BM and blood represent the common locations for myeloma cells, they can also expand and survive outside of the BM, particularly, in soft tissue and visceral organs, leading to aggregates of malignant plasma cells termed as plasmacytoma [2, 3]. Myeloma commonly presents with symptoms and signs of end‐organ damage related to the clonal plasma cell expansion in the BM (bone destruction leading to lytic lesions/fractures, hypercalcemia, anemia, and marrow‐infiltrating lesions) or related to the monoclonal protein (renal dysfunction). During the past two decades, the therapeutic options for myeloma have expanded significantly with the introduction of over a dozen new drugs representing different drug classes with consequent improvement in survival. The median survival of patients with newly diagnosed myeloma has nearly tripled over this time period, but with the disease remaining mostly incurable, most patients go through multiple rounds of treatment and eventually become refractory to the available therapeutics [3, 4]. During the disease course, there has been an increasing incidence of plasma cells expanding outside of the BM, likely a reflection of increasing independence of the clonal plasma cells from the BM microenvironment, which is often termed as extramedullary disease (EMD) [2, 3, 5, 6]. EMD may take the form of solid lesions, or extramedullary plasmacytomas, in a variety of locations or present as increasing numbers of plasma cells in the peripheral circulation (plasma cell leukemia [PCL]) [3]. EMD may be present at initial diagnosis though it is more common when patients have relapsed or become refractory to treatment [7]. True EMD that is independent of bone is clinically distinct from MM in, or adjacent to, BM [3]. It is characterized by high‐risk genetic features, resistance to therapy, and poor prognosis, and is considered a factor for high risk of progression or relapse in MM [3, 8, 9, 10]. Recent studies have highlighted the poor prognosis of patients with EMD, particularly, in the context of relapsed or refractory disease, with even highly effective treatments in MM associated with limited responses in this subgroup [11].

## 2 Definitions

**FIGURE 1:**
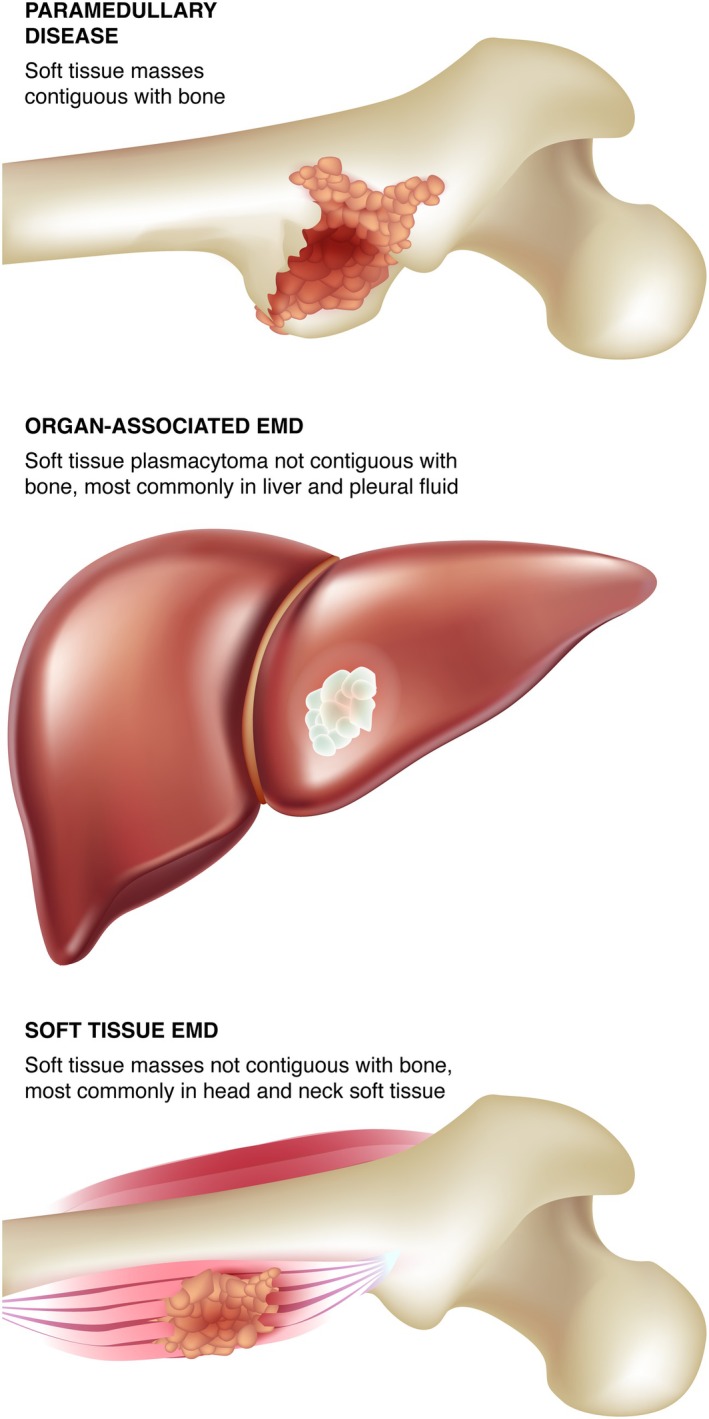
EMD may erupt from bone marrow (PMD) or spread to organs and/or soft tissues independent of bone. [Color figure can be viewed at wileyonlinelibrary.com]

Broadly, true EMD reflects identification of clonal plasma cell growth outside of the BM microenvironment, such as soft tissue, visceral organs, central nervous system (CNS), or blood [12]. True EMD should be clearly distinguished from paramedullary disease (PMD), which refers to the contiguous expansion of marrow plasma cells adjacent to the bone through cortical destruction associated with lytic bone disease (Figure 1). PMD does not reflect the aggressive biology of true EMD [3, 13]. EMD can be broadly classified based on disease location as soft tissue plasmacytoma, including diffuse organ infiltration, CNS myeloma (CNS‐MM), or PCL. EMD can be present either at initial diagnosis (primary EMD) or at relapse (secondary EMD) [9, 10].

### 2.1 Soft Tissue Plasmacytoma and Visceral Organ Infiltration/Plasmacytoma

These plasmacytomas can affect virtually any soft tissue, with muscle, skin, and the respiratory or gastrointestinal tract being some of the most common locations [2, 14]. Visceral organs such as the liver and spleen may be diffusely infiltrated by malignant plasma cells, or may develop solid plasmacytomas that can be visualized in individual organs. At diagnosis, the head and neck have been reported as the most common locations, with liver and pleural fluid the most common locations at relapse [15].

### 2.2 CNS‐MM

This plasmacytoma is relatively rare and may present with clonal plasma cells in the cerebral spinal fluid, accompanied by meningeal infiltration and intraparenchymal plasmacytomas [16]. This is often classified as a distinct entity due to its prognostic significance as well as the unique treatment approaches geared toward penetrating the blood–brain barrier [17]. Although CNS‐MM is an unmet need due to its rare and aggressive nature [18], we will not review this form of MM further in this article.

### 2.3 PCL

Historically, this aggressive variant of MM was defined by the presence of circulating plasma cells (cPCs; > 20% and/or absolute count > 2 × 10^9^/L) [19, 20]. However, this criterion was recently revised to ≥ 5% cPCs or an absolute count ≥ 0.5 × 10^9^/L, as detected morphologically on a peripheral blood smear, given similar outcomes among patients with lower counts [20]. The clinical presentation of PCL is more aggressive than that of MM, including more severe cytopenias, hypercalcemia, and renal insufficiency, reflecting higher tumor burden and proliferation [19]. Extramedullary involvement (lymph nodes, liver, spleen, pleura, and CNS) at diagnosis is more common with PCL than MM [19, 20].

It is also important to distinguish solitary plasmacytoma (SP) with < 10% plasma cells in the BM or SP without BM involvement [12]. These conditions do not fulfill the criteria for symptomatic myeloma and thus are not considered EMD. Only a fraction will progress to symptomatic myeloma, more commonly when clonal plasma cells are seen in the marrow [21].

## 3 Epidemiology

Some studies include patients with either true EMD or bone‐contiguous PMD, resulting in heterogeneous populations and outcomes [3]. However, the overall incidence of true EMD is more rare (0.5%–5.2%) at diagnosis, increasing to approximately 5%–30% among different series in the setting of relapsed disease [7]. For PMD, the incidence at diagnosis (7%–32%) is similar to the incidence at relapse (10%–30%). With a higher incidence of EMD in relapsed disease, studies over the years have also noted an increasing overall incidence of EMD; in one study, nearly 6.5% in 2005 had EMD compared with 23.7% in 2014 [22]. It is likely that the increasing frequency of true EMD among patients with MM reflects the improved survival with the advent of newer therapies and the tendency for today's patients to have more advanced disease, along with improvement in imaging techniques, such as computed tomography (CT), positron emission tomography (PET)‐CT, and magnetic resonance imaging (MRI) [23]. The timing of emergence also differs between EMD and PMD. Median time from diagnosis to emergence of secondary EMD has been reported to be 19–23 months [24, 25], and the median age for patients with PMD is nearly 10 years higher compared with patients who have true EMD [25].

Like EMD, PCL can be identified at diagnosis (primary PCL, 0.6% of MM cases), or more commonly, at the time of disease relapse (secondary PCL) with a median time from MM diagnosis to leukemic progression of ~20–22 months [26, 27].

## 4 Risk Factors

Various baseline and treatment‐related characteristics have been associated with increased incidence of EMD. Data from the total therapy protocol trials reported that extramedullary involvement at presentation was more common among those with high‐risk translocations *t*(14;16) and *t*(14;20) [28]. The presence of high‐risk cytogenetics at baseline, as well as the development of new high‐risk genetic lesions, such as 17p deletion and 1q abnormalities, has been associated with the development of secondary EMD [29]. A few studies have reported the association of high‐risk cytogenetics, such as *t*(4;14), *t*(14;16), gain(1q21), and del(17p), in patients with EMD, with at least one high‐risk abnormality present at diagnosis in over half of patients with EMD [25, 29, 30, 31, 32, 33]. Studies have also identified del(17p13) and del(13q14) as markers for progression to EMD and del13 as a risk factor for EMD relapse [25, 34]. In a study of 234 patients with secondary EMD from the Czech Republic, younger age, high lactate dehydrogenase (LDH) levels, extensive osteolytic activity, and immunoglobulin A or the nonsecretory type of MM at the time of MM diagnosis were identified to be the main risk factors for secondary EMD development [35]. Other significant risk factors include therapeutic history (> 2 lines of treatment ± treatment duration > 6 months) and allogeneic stem cell transplant (allo‐SCT) [29, 36, 37].

## 5 Pathogenesis

Although the mechanistic drivers of EMD remain incompletely understood, it is evident that EMD is complex and more heterogeneous than MM [7, 38]. Several key mechanisms that have been implicated in its pathophysiology include alterations in chemokine receptors and adhesion molecules, genetic and epigenetic changes, and substantial heterogeneity both within the tumors and their microenvironment.

### 5.1 Chemotaxis and Adhesion Molecules

Evidence suggests that the development of EMD occurs after MM cells lose anchorage and adhesion to the BM, allowing cells to migrate, proliferate, and survive outside the BM [7]. Studies have implicated a loss of adhesion and disruption of signals regulating migration. Chemotaxis and adhesion of myeloma cells to the BM are mediated by signaling cascades between the myeloma cells and the BM microenvironment, including the binding of stromal‐derived factor 1α to the CXCR4 receptor and adhesion molecules such as VLA‐4, P‐selectin, CD56, and CD44 [2, 37, 39]. Tumor dissemination from the BM may be related to reduced expression of chemokine receptors and adhesion molecules, underexpression of membrane‐embedded CD81/CD82 tetraspanins, and overexpression of the tumor‐promoting heparanase enzyme [12, 40]. Another proposed mechanism is an upregulation of CXCR4 by various growth factors and hypoxic conditions in the tumor microenvironment [41], as well as the acquisition of the EMD phenotype regulated by CXCR4 [41, 42, 43]. Other pathways, such as the PCAT‐1/Wnt β‐catenin signaling axis, have also been implicated in the growth, survival, and migration of MM cells outside of the BM [44, 45].

### 5.2 Genetic and Epigenetic Changes

In addition to loss of adhesion, aggressive disease features driven by genetic and epigenetic changes are more prevalent in patients with EMD. Acquired abnormalities typically seen in myeloma such as del(17p) and 1q abnormalities are more prevalent in EMD tumor cells than in MM cells in the BM [46, 47]. Although no unifying genetic event has been identified for EMD in whole‐genome sequencing, recent studies have noted an increased prevalence of RAS–RAF mutations in EMD cells, implicating the MAPK pathway [48]. Frequently identified oncogenes included BRAF, NRAS, KRAS, and MYC, as well as loss of tumor suppressor genes SP140 and TENT5C. Studies also suggest that MAPK‐activating mutations may co‐occur with cytogenetic abnormalities. One such study showed that RAS/BRAF mutations are likely essential for the development of EMD and suggested that the underlying reason for negative outcomes in patients with poor prognostic factors, such as duplication of 1q and deletion of 17p, is largely due to EMD development [8]. The study also suggested that the mutation sites of TP53 were different between EMD versus non‐EMD patients, with gain‐of‐function mutations enriched in patients with EMD. In another study involving 14 EMD samples, the co‐occurrence of 1q21 gain/amplification and MAPK pathway mutations in 79% of samples suggested that these may be crucial mutational events in EMD development [49]. It was also observed that patients with mutated KRAS and 1q21 gain/amplification at the time of diagnosis have a significantly higher risk of EMD development in an analysis using the CoMMpass dataset [37, 49]. Downregulation of CXCR4 and enhanced cell proliferation, along with reduced expression of therapeutic targets (CD38, SLAMF7, GPRC5D, FCRH5), were demonstrated in this study [49].

### 5.3 EMD and the Tumor Microenvironment

Cytogenetic abnormalities alone may not explain the spread of EMD [50]. Emerging evidence suggests that EMD may exhibit a complex, solid tumor‐like architecture characterized by spatial and temporal heterogeneity of the microenvironment [51, 52]. In one recent study exploring spatial transcriptomics in EMD biopsies, substantial intratumoral and intrapatient heterogeneity was observed at the copy number level within plasma cells, including the emergence of new subclones in circumscribed areas of the tumor, highlighting the genomic instability present in spatially distinct areas of the tumors [51]. Analysis of EMD sections revealed intratumoral expression differences in GPRC5D and B‐cell maturation antigen (BCMA; also known as TNFRSF), two important target antigens for bispecific antibody therapy.

Contrary to previous perceptions, EMD lesions have been found to be infiltrated by various immune cells, including T cells [51, 52]. Interestingly, exhausted TIM‐3+/PD‐1+ T cells were found to diffusely colocalize with MM cells, whereas functional and activated CD8+ T cells and M1 macrophages showed a focal infiltration pattern in tumor‐free regions [51, 52]. The observation that active T cells could be spatially confined to niches segregated from MM cells in EMD lesions suggests that therapies such as targeted T‐cell redirectors may infiltrate the lesions and eradicate MM cells [51, 52].

### 5.4 MicroRNA Analysis

MicroRNAs are increasingly recognized for their role in the development and progression of MM, including EMD. Some studies have identified differences in long, noncoding RNA microRNAs, the proteome of BM mononuclear cells, and the metabolite signature in plasma between patients with EMD and those without [53, 54, 55]. Long, noncoding RNA, such as MALAT1, can regulate gene expression and may play a role in tumor initiation, metastasis, and drug resistance [56]. MALAT1 was shown to be markedly higher in EMD cells compared with intramedullary MM cells, and patients with a greater reduction in MALAT1 after initial treatment had significantly longer progression‐free survival (PFS) than those with minimal change following treatment [56].

In a microRNA analysis, 42 microRNAs were significantly dysregulated in the BM of patients with MM, EMD, and PCL; subsequent validation by RT‐qPCR confirmed elevated levels of miR‐140‐3p, miR‐584‐5p, miR‐191‐5p, and miR‐143‐3p in patients with MM compared to patients with EMD and PCL [54]. Another study used label‐free mass spectrometry and plasma proteomics on BM mononuclear cells and plasma from patients with and without EMD to identify three proteins that were promising markers of EMD for potential therapeutic targeting [55]. These were vascular cell adhesion molecule 1 (VCAM‐1), pigment epithelium‐derived factor (PEDF), and hepatocyte growth factor activator (HGFA). Metabolomic analysis revealed a distinct metabolite signature in the plasma of EMD patients compared with the plasma of patients with MM, suggesting metabolic rewiring in EMD tissue that could potentially open avenues for therapeutic intervention [55].

While research is improving our understanding of EMD, a common finding is that EMD is more complex and heterogeneous than MM, with significant inter‐ and intrapatient heterogeneity [7]. While no single biomarker for EMD biology has been identified, different studies have implicated various mutations in the MAPK pathway, abnormalities in 1q and loss of *TP53*, reduced expression of chemokine receptors, adhesion molecules, and certain therapeutic targets, and enrichment of exhausted T cells in the development of disease [7, 8, 12, 33, 48, 51]. Increased heterogeneity and complexity are reflected in the poor outcomes of patients with EMD even as novel, effective MM therapies have been introduced [2, 3, 7].

## 6 Diagnostic Approach

Diagnosis of EMD relies on sensitive and specific imaging modalities and, at times, proven detection of clonal plasma cells through directed biopsies. International Myeloma Working Group (IMWG) criteria have now been updated so that detection of > 1 lytic lesion assessed by CT, whole‐body low‐dose CT, or PET‐CT, and > 1 focal BM lesion (≥ 5 mm) on MRI, is sufficient to establish bone disease [57, 58]. With the use of sensitive imaging techniques, including MRI and PET‐CT, EMD may be found in up to 30% of patients with MM across the overall disease course. PET‐CT (typically using ^18^F‐fluorodeoxyglucose [FDG]) and whole‐body MRI with diffusion‐weighted imaging (DWI) are potentially the most effective modalities for identifying extramedullary tumor sites [59, 60, 61]. PET‐CT combines the functional assessment of the metabolic activity of nearby cells with the morphology assessment of PET, allowing it to detect and distinguish between metabolically active and inactive sites of clonal proliferating plasma cells [60, 62, 63]. It is recommended for cases of suspected soft tissue involvement [14]. Whole‐body MRI with DWI combines data on cellular density from the microscopic motion of water molecules within tissues with the morphology assessment of MRI and is the preferred modality in cases of suspected CNS involvement and focal/diffuse BM lesions [14, 62].

In addition to their role in diagnosis ^18^F‐FDG PET‐CT and DWI are effective modalities for evaluating and monitoring response to treatment [59, 60, 62]. PET‐CT measures FDG uptake, which allows quantification of changes in disease burden over time, and DWI assesses the apparent diffusion coefficient relative to cell density. The same imaging modality should be used consistently before and after therapy to ensure accurate evaluation [64, 65]. In cases in which PET‐CT shows no metabolic activity in the PET component but residual disease is suspected on the CT component, DWI MRI should be used to evaluate response [57, 62, 66].

Along with the imaging workup, a routine myeloma workup includes monoclonal protein studies, serum LDH, and a BM biopsy to evaluate the degree of total plasma cell infiltration [21, 61]. Elevated levels of LDH are often observed in the context of EMD [24]. A lesion biopsy should be considered when imaging or diagnosis is unclear, guided by a careful risk–benefit assessment, factoring in age, performance status, therapeutic impact, and lesion accessibility [67]. It may be most appropriate in fit patients, particularly when the biopsy result may influence clinical decision making [67].

Confirmation of malignant plasma cells by hematopathology, including immunophenotyping, reveals distinct profiles in EMD, characterized by lack of CD38 expression compared with BM lesions, discordant CD44 and CD56 expression, higher proliferative index, lower p27 expression, CCND‐1 positivity, strong BCl‐2 and Bcl‐xl expression, CD56 downregulation, and CD44 up‐regulation [68, 69, 70].

Patients who develop EMD during their disease course often have relatively low levels of serum M protein and/or serum free light chains, reflecting a shift toward oligosecretory disease, and are typically excluded from clinical trials [6, 71]. IMWG/International Myeloma Society (IMS) response criteria are being refined to diminish the need for urine testing and update the role of PET‐CT, which may allow more patients with EMD to meet inclusion criteria for clinical trials [72]. The presence of circulating tumor DNA may offer another avenue for monitoring patients whose disease is nonsecretory [73]. A recent study explored the use of mass spectrometry on peripheral blood as a screening tool for the presence of EMD in newly diagnosed MM [74]. Investigators demonstrated that MALDI‐TOF mass spectrometry fingerprinting of peripheral blood can distinguish between patients with MM and patients with primary EMD. The analysis suggested that the method predicts specifically primary EMD with high sensitivity (86.4%), accuracy (78.4%), and specificity (72.4%) [74].

## 7 Natural History

Presence of EMD is a powerful negative prognostic factor in MM, with the relatively poor outcomes of such patients well described in multiple studies [75, 76, 77, 78, 79, 80, 81]. Survival is typically less than 2–3 years following the development of EMD during the disease course, and overall survival (OS) is consequently significantly shorter in patients with EMD compared to those without EMD [28, 82]. Factors associated with inferior outcomes among those with EMD include secondary EMD, multiple organ involvement, CNS involvement, poor response to therapy, β_2_‐microglobulin > 5 mmol/L, and International Staging System (ISS) Stage III disease [81, 82, 83, 84, 85]. In a retrospective analysis of 120 patients with EMD treated between 2007 and 2022, the partial response or better (≥ PR) rate to initial treatment was 67%, and the rate of imaging response was 59% [86]. With a median follow‐up of 84.2 months, the median PFS and OS were 10.5 and 24.5 months, respectively, with longer survival for those with primary EMD (20.2 and 34.5 months, respectively) than secondary EMD (5.8 and 12.4 months, respectively). Secondary EMD and organ involvement were found to be negative prognostic factors of both PFS and OS [86]. In a study of 371 patients with EMD, comprising 113 patients with de novo EMD and 258 patients with EMD at relapse, LDH levels > 250 U/L, creatinine levels > 177 μmol/L, and ≥ 2 high‐risk genetic abnormalities were identified as independent adverse prognostic factors [87].

## 8 Treatment Approaches

Given the relatively infrequent occurrence of EMD, especially in the past, there is limited prospective data to support specific treatment approaches [67]. Clinical practice has largely been guided by developments in the treatment of MM and data from small retrospective EMD cohorts that have been reported over time. Some of the evidence for standard regimens (including conventional chemotherapy, immunomodulatory drugs [IMiDs], proteasome inhibitors, anti‐CD38 monoclonal antibodies [mAbs], and selinexor) used in patients with EMD are shown in Table 1 [14]; these data highlight the inconsistent definitions applied, the management of those with EMD as a subgroup receiving similar treatments to the overall group, and the relatively few patients with EMD historically enrolled in studies. Despite these limitations, consistently poor outcomes were observed for patients with EMD across the studies. In patients with NDMM and true EMD, most have been shown to progress within 1–3 years following treatment with multiple SOC regimens and transplant therapies. In patients with heavily pretreated RRMM and EMD, the poor outcomes with standard therapies were further elucidated in LocoMMotion and MoMMent, the noninterventional studies of real‐world standard regimens. Overall response rate (ORR) in these studies was lower in patients with true EMD compared with those without EMD (24.1% vs. 32.5%) and median PFS and OS were also shorter (2.7 vs. 4.6 and 7.2 vs. 14.7 months, respectively) [11]. In a meta‐analysis of patients receiving standard regimens for EMD in clinical trials, including anti‐CD38‐based regimens, the pooled ORR for the subgroup with true EMD was lower compared with those who did not have EMD (20.7% vs. 66.2%), with shorter PFS and OS (6.3 vs. 12.9 and 21.0 vs. 39.0 months, respectively) [102]. The analysis suggested that patients with EMD had 87% worse chance of response (hazard ratio 0.13 [95% CI 0.09–0.20]) than those without [102].

**TABLE 1:** Outcomes in patients with EMD with standard therapies [10, 22, 33, 81, 88, 89, 90, 91, 92, 93, 94, 95, 96, 97, 98, 99, 100, 101].

Regimen	Disease setting	Total patients	No. of patients and type of EMD	Study type	True EMD outcomes	Outcomes in other groups
**Dara‐VCd** [101]	NDMM and 1st relapse	40	22 True EMD 14 PMD 4 Both	Prospective Phase 2 trial	—	**All EMD** ORR: 80% mPFS (NDMM): 26 months mPFS (relapse): 15 months
**Multiple regimens** [97] (ASCT, MP, Rd, Td, Vd, VMP, VTd, or high‐dose dex)	NDMM	64	22 True EMD 42 PMD	Retrospective (2009–2016)	**True EMD:** mPFS: 16 months 2‐year OS: 35.1%	**PMD:** mPFS: 16.1 months 2‐year OS: 52.6%
**Multiple regimens** [81]	NDMM and at relapse	130 NDMM 96 at relapse	NDMM: 92 True EMD 38 PMD At relapse: 84 True EMD 12 PMD	Retrospective (2010–2017)	**True EMD (NDMM)** mPFS: 38.9 months mOS: 46.5 months **True EMD (relapse)** mPFS: 13.6 months mOS: 11.4 months	**PMD (NDMM)** mPFS: 51.7 months mOS: Not reached **PMD (relapse)** mPFS: 20.9 months mOS: 39.8 months
**Autologous transplant** [33]^a^	NDMM	488	87 True EMD only 374 PMD only 27 Both	Registry analysis (2003–2015)	**True EMD only:** 4‐year PFS: 39% 4‐year OS: 60%	**PMD only:** 4‐year PFS: 44% 4‐year OS: 72%
**Autologous transplant** [22]^a^	NDMM	682	139 True EMD 543 PMD	Registry analysis (2005–2014)	**True EMD:** 3‐year PFS: 39.9% 3‐year OS: 58.0%	**PMD:** 3‐year PFS: 50.0% 3‐year OS: 77.7%
**IMiD and/or PI‐based regimens** [98]	NDMM	2,332	12 True EMD 243 PMD 12 not classified	Meta‐analysis of 8 trials	—	**All EMD (*n* = 267)** mPFS: 25.3 months mOS: 63.5 months **All non‐EMD** mPFS: 25.2 months mOS: 79.9 months
**Benda‐PD** [89]	RRMM (median 4 prior LOT)	11	5 True EMD 6 PMD	Retrospective (2019–2022)	—	**All EMD** ORR: 54% 2‐year PFS: 71.3% 2‐year OS: 81.8%
**PomIxaDex** [100]	RRMM and PCL	17	11 True EMD 6 PCL	Prospective Phase 2 trial	—	**All patients** ORR: 35% mPFS: 4.5 months
**Dexa‐Beam** [91]	RRMM (mean 3 prior LOT)	18	11 True EMD	Retrospective (2007–2012)	**True EMD** ORR: 54.5% mPFS: 4 months	**Non‐EMD** ORR: 42.9% mPFS: 3 months
**VD‐PACE + IMiD** [90]	RRMM (median 3 prior LOT)	21	21 EMD based on 2014 IMWG criteria	Retrospective (2012–2020)	—	**All EMD** ORR: 76% mPFS: 5.5 months mOS: 14.9 months
**Dara‐DCEP** [95]	RRMM (median 3 prior LOT)	32	23 True EMD 9 PMD	Prospective Phase 2 trial	**True EMD** ORR: 39.1%	**PMD** ORR: 25.0% **All EMD** ORR:67.7% mPFS: 5 months mOS: 10 months
**D(T)‐PACE** [88]	RRMM (median 2 prior LOT)	43	43; EMD type undefined	Retrospective (2006–2018)	—	**All EMD** ORR: 58% mPFS: 5.0 months mOS: 9.0 months
**Carfilzomib‐based regimens** [93]	RRMM (median 4 prior LOT)	45	25 EMD 20 PMD	Retrospective (2013–2019)	—	**All EMD** ORR: 59% mPFS: 5 months mOS: 10 months
**CED** [92]	RRMM (median 4 prior LOT)	70	35; EMD type undefined	Retrospective (2014–2022)	**EMD** ORR: 51%	**All patients** ORR: 52% mPFS: 6.2 months mOS: 10.9 months
**Elo‐based combination therapy** [96]	RRMM (median 4 prior LOT)	74	15 with evidence of infiltration with aberrant plasma cells in tissue biopsies from extraosseous sites	Retrospective (2016–2020)	—	**All EMD** ORR: 40% mPFS: 3.8 months mOS: 12.9 months
**Selinexor‐dex** [99]	RRMM (triple‐class refractory)	122	22 True EMD 5 PMD	Subgroup analysis of a Phase 2 trial	—	**All EMD** ORR: 18.5%
**Pom + Dex** [10]	RRMM (median 6 prior LOT)	174	13 True EMD	Retrospective (2007–2010)	**True EMD** ORR: 31% mOS: 16 months	**Overall** mOS: Not reached
**Dara monotherapy and DRd** [94]	RRMM (Dara: median 5 prior LOT; DRd: median 1 prior LOT))	41 Dara 186 DRd	14 True EMD and PMD (Dara) 10 True EMD (DRd) 31 PMD (DRd)	Retrospective (2016–2019)	**True EMD (DRd)** mPFS: 4.8 months	**All EMD (Dara)** ORR: 21.4% mPFS: 1.4 months mOS: 4.6 months **Non‐EMD (Dara)** ORR: 50.0% mPFS: 6.2 months mOS: 15.4 months **All EMD (DRd)** ORR: 57.7% mPFS: 7.8 months **Non‐EMD (DRd)** ORR: 85.4% mPFS: 27.3 months **PMD (DRd)** mPFS: 8.4 months

*Note*: Bolding is used to separate the groups.

Abbreviations: ASCT, autologous stem cell transplant; Benda, bendamustine; CED, cyclophosphamide, etoposide, and dexamethasone; D(T), dexamethasone ± thalidomide; Dara, daratumumab; DCEP, dexamethasone, cyclophosphamide, etoposide, and cisplatin; dex, dexamethasone; Dexa‐Beam, dexamethasone, carmustine, cytarabine, etoposide, and melphalan; DRd, daratumumab, lenalidomide, and dexamethasone; Elo, elotuzumab; EMD, extramedullary disease; IMiD, immunomodulatory drug; IMWG, International Myeloma Working Group; LOT, line of therapy; MP, melphalan and prednisone; NDMM, newly diagnosed multiple myeloma; ORR, overall response rate; OS, overall survival; PomIxaDex, pomalidomide, ixazomib, and dexamethasone; PACE, cisplatin, doxorubicin, cyclophosphamide, and etoposide; PCL, plasma cell leukemia; PD, pomalidomide and dexamethasone; PFS, progression‐free survival; PMD, paramedullary disease; Pom, pomalidomide; Rd, lenalidomide and dexamethasone; RRMM, relapsed/refractory multiple myeloma; Td, thalidomide and dexamethasone; VCd, bortezomib, cyclophosphamide, and dexamethasone; Vd, bortezomib and dexamethasone; VMP, bortezomib, melphalan, and prednisone; VTd, bortezomib, thalidomide, and dexamethasone.

^a^ The 2 autologous transplant studies may have overlapping patient data from the registry.

### 8.1 Standard Regimens

Although outcomes for patients with EMD are poor compared with non‐EMD MM, newer agents (e.g., lenalidomide and bortezomib‐based regimens) have shown improved response rates compared with conventional chemotherapy in patients with de novo EMD [83, 88, 103]. Regimens that combine these agents with traditional cytotoxic chemotherapy may be effective in controlling disease [89, 90, 104, 105]. In relapsed patients with EMD, polychemotherapy regimens such as cisplatin, doxorubicin, cyclophosphamide, and etoposide (PACE); dexamethasone, carmustine, cytarabine, etoposide and melphalan (Dexa‐BEAM); and hyperfractionated cyclophosphamide, vincristine, doxorubicin, and dexamethasone (HyperCVAD) followed by autologous stem cell transplant (ASCT) or allo‐SCT have been successful (Table 1) [91, 105]. Other combination regimens (e.g., cyclophosphamide, etoposide, and dexamethasone) have shown efficacy in the relapsed setting [92]. Although patients may benefit from aggressive chemotherapy‐based approaches, the duration of response and OS remain poor [2, 91, 92]. As a result, chemotherapy is commonly used as a cytoreducing bridging therapy before CAR‐T therapy, where it has been shown to improve outcomes in patients with EMD who receive CAR‐T [106, 107]. Newer generation IMiDs, such as pomalidomide, have been effective in some patients at relapse, and carfilzomib‐based treatment strategies have shown some efficacy in heavily pretreated patients with extramedullary RRMM [10, 93, 108].

More recently, several studies have explored the role of daratumumab in the management of EMD, with some reporting limited efficacy [94]. Analysis of patients with EMD or PMD in the prospective phase 2 EMN19 study of DVCd showed a median PFS of 26 months for patients with NDMM and 15 months for patients treated at first relapse. An updated pooled analysis of studies (GEN501 part 2 and SIRIUS) evaluating the efficacy of daratumumab in heavily pretreated patients reported an ORR of 16.7% (95% CI 3.6–41.4) in a subset of patients with EMD, with improved OS in responders versus those with minimal response/stable disease [109]. In an Italian multicenter observational analysis of 102 patients with EMD, treatment with daratumumab‐based regimens was associated with high rates of biochemical and imaging responses at diagnosis, with lower rates at relapse [110]. In a Phase 2 trial of 32 patients, daratumumab was combined with dexamethasone, cyclophosphamide, etoposide, and cisplatin (Dara‐DCEP), and the ORR was 67.7% with best response complete response or better (≥ CR) in 35.5% [95]. The median PFS was 5 months and median OS was 10 months. Elotuzumab, a mAb targeting SLAMF7, has also been studied in this setting [96, 111].

Studies in small cohorts receiving newer therapies, such as selinexor, have demonstrated potential efficacy, warranting further study [112]. Reports have suggested the efficacy of venetoclax‐based regimens in patients with EMD and *t*(11;14) [113], and agents targeting specific mutations such as B‐Raf v600E have shown benefit in some reports when tumor cells carry these lesions [114].

### 8.2 Stem Cell Transplant

The benefit of ASCT in patients with EMD has been studied extensively [22, 83]. The Spanish PETHEMA group observed a significantly shorter median OS (46.7 months vs. not reached [NR]) in patients with EMD versus those without EMD, but no significant difference in 2‐year PFS after ASCT with high‐dose melphalan conditioning [2]. Single vs. multiple sites of EMD as well as organ involvement can also impact prognosis after ASCT [2, 22]. Upfront tandem transplant has been shown to overcome poor outcomes in these patients compared to single ASCT [2, 33]. Studies evaluating tandem transplantation suggest high‐risk subgroups, including patients who fail to achieve very good partial response (VGPR) after single ASCT, those with ISS Stage II/III disease, and patients with high‐risk cytogenetics, may benefit most from tandem transplantation [2, 115, 116]. However, a European Society for Blood and Marrow Transplantation (EBMT) registry study reported similar 3‐year PFS and OS with both first‐line tandem and single ASCT in patients with EMD [117]. Presence of EMD has been associated with inferior PFS after ASCT with a significant increase in early progression even in patients on maintenance therapy [85].

The role of allo‐SCT remains unclear due to its association with high transplant‐related mortality and graft‐versus‐host disease. Additionally, many patients undergoing allo‐SCT, particularly with reduced‐intensity conditioning aimed at reducing nonrelapse mortality, may still relapse with EMD [84].

### 8.3 Novel Immunotherapy Agents

Data from observational studies suggest that response rates to standard therapies are typically below 25% in patients with RRMM and EMD, with median PFS and OS of just 2.7 and 7.2 months, respectively [11]. Innovative approaches using immunotherapies including ADCs, adoptive cell therapy (chimeric antigen receptor [CAR] T cells), and, bispecific antibodies have shown promising results in a limited number of relapsed patients with EMD (Table 2) [29, 118, 124, 125, 126, 127, 128, 129, 130].

**TABLE 2:** Response rates with novel immunotherapies in patients with RRMM [107, 118, 119, 120, 121, 122, 123].

Therapy	EMD in real‐world studies of CAR‐T				EMD in clinical trials of ADCs and bispecific antibodies								
	Ide‐cel [118]		BCMA CAR‐T [107]		Belantamab mafodotin [123]		Teclistamab [119]		Elranatamab [120]		Talquetamab [122]^a^		Teclistamab + Talquetamab [121]
EMD type and comparator	True EMD^b^ (*n* = 84)	Non‐EMD (*n* = 267)	True EMD^c^ (*n* = 47)	Non‐EMD (*n* = 105)	EMD undefined (*n* = 22)	Total population (*n* = 97)	True EMD^d^ (*n* = 28)	Non‐EMD (*n* = 165)	True and paramedullary EMD^e^ (*n* = 39)	Non‐EMD (*n* = 84)	True EMD^d^ (*n* = 154)	Non‐EMD (*n* = 41)	True EMD^f^ (*N* = 90)
ORR	52%	82%	58%	96%	5%	32%	35.7%	68.6%	38.5%	71.4%	41.4	69%	79%

Abbreviations: ADC, antibody‐drug conjugate; BCMA, B‐cell maturation antigen; CAR, chimeric antigen receptor; EMD, extramedullary disease; ide‐cel, idecabtagene vicleucel; ORR, overall response rate; RRMM, relapsed/refractory multiple myeloma. Shaded columns report data from patients with true EMD.

^a^ 0.8 mg/kg biweekly dose.

^b^ Patients with both true EMD and paramedullary EMD were classified as true EMD, and patients with paramedullary EMD only were classified as non‐EMD.

^c^ Defined as bone‐independent (only) tumors of plasma cells growing at anatomical sites outside of the bone marrow detected within 30 days of CAR T‐cell infusion.

^d^ Defined as soft tissue plasmacytomas that were not associated with bone.

^e^ Defined as the presence of any plasmacytoma (extramedullary and/or paramedullary with a soft tissue component).

^f^ ≥ 1 nonradiated bone‐independent soft tissue plasmacytoma ≥ 2 cm in greatest dimension confirmed by central review of PET‐CT scans or whole‐body MRI (with sponsor approval).

BCMA‐targeting ADCs have demonstrated encouraging activity in a limited number of cases of relapsed EMD [129]. Outcomes with BCMA‐targeted CAR‐T therapy have moreover shown an improvement over standard, non‐T‐cell redirecting approaches for patients with EMD; however, outcomes are inferior compared with those of patients with no EMD. In a retrospective study of 152 patients receiving standard‐of‐care CAR‐T therapy, only 47 (31%) had true EMD [107]. The true EMD group had a higher incidence of high‐grade cytokine release syndrome (CRS), steroid and anakinra use, and thrombocytopenia 30 days after infusion compared with those without EMD. In addition, patients with EMD had an inferior ORR (58% vs. 96%), median PFS (5.1 vs. 12.4 months), and OS (12.2 vs. 27.5 months). In contrast, patients with PMD had similar median PFS and OS compared with those with MM restricted to the BM. Notably, among the 189 patients identified in this study, 14 did not receive CAR‐T therapy due to disease progression or death and 23 were pending infusion at data cut‐off, highlighting that off‐the‐shelf options will be needed for some patients [107].

Another recent study examined the outcome of patients with true EMD in RRMM treated with idecabtagene vicleucel (ide‐cel). Among 351 patients, 84 (24%) had EMD before infusion [118]. ORR at Day 90 was 52% versus 82% for the EMD and non‐EMD cohorts, and the median PFS was 5.3 months for the EMD cohort versus 11.1 months for the non‐EMD cohort. The median OS was 14.8 versus 26.9 months for the EMD and non‐EMD cohorts, respectively. EMD was an independent predictor of inferior responses and survival in this study. In a retrospective study of 134 patients treated with CAR‐T cell therapy, those with true EMD had shorter PFS (9.0 vs. 24.2 months) and OS (24.0 months vs. NR) compared with those with no EMD or PMD [13]. Half of the patients who relapsed in this study had EMD at relapse [13]. An exhausted T‐cell profile prior to CAR‐T therapy has been associated with poor outcomes in this setting [125]. Based on small studies, it has been suggested that combination approaches, such as coadministration of pomalidomide with CAR‐T therapy may be associated with increased responses in EMD [131]. However, a significant unmet need remains, as CAR‐T therapy often requires weeks for cell manufacturing, a delay that many patients with aggressive EMD cannot tolerate. Furthermore, CAR‐T therapy remains inaccessible in many regions [132].

A similar pattern of worse outcomes with EMD was observed in clinical studies of bispecific antibodies in patients with RRMM. In a clinical study of teclistamab, a BCMAxCD3 bispecific antibody, patients with true EMD had a lower ORR (35.7% vs. 68.6%) than those without [119]. Similarly, a clinical study of elranatamab showed a lower response rate (38.5% vs. 71.4%) and a lower probability of maintaining response at 15 months (70.6% vs. 77.9%) in patients with EMD (true EMD and/or PMD) compared with those without [120]. Finally, patients with true EMD also had lower response rates across two dosing cohorts with the GPRC5DxCD3 bispecific antibody talquetamab (41.4%–48.5% vs. 79.6%–81.2%) compared with those who did not have EMD [133].

Targeting both BCMA and GPRC5D antigens could hypothetically increase the number of bound antibodies per tumor cell, due to the heterogeneous epitope expression observed in EMD samples [134]. A recent Phase 1 trial (RedirecTT‐1) evaluating the BCMA‐targeted bispecific antibody teclistamab and the GPRC5D‐targeted bispecific antibody talquetamab reported promising outcomes in the subgroup of patients with true EMD [134]. This led to a large dedicated Phase 2 study in 90 patients with true EMD that demonstrated an ORR of 79%, nearly double the ORR of the respective monotherapies [119, 121, 122]. It is one of the first studies to have prospectively assessed EMD response using central radiology review of whole‐body PET‐CT [121]. The responses in RedirecTT‐1 were also durable over 1 year in most patients, with 12‐month duration of response, PFS, and OS rates of 64% and 61%, and 74%, respectively [121]. These efficacy results surpass any other therapy for patients with EMD [10, 88, 89, 90, 91, 92, 93, 95, 96, 121].

### 8.4 Radiation Therapy (RT)

There is no consensus on the use and impact of RT in EMD. RT targets tumors in MM by inducing apoptosis, decompressing nervous structures, reducing inflammation, inhibiting osteoclasts, and promoting bone healing; however, the potential sensitization of normal tissues remains a concern [135]. RT is most often employed to control symptomatic lesions in the context of systemic therapy [135]. However, there is some evidence of synergy of RT with CAR‐T therapy or checkpoint inhibitors via the abscopal effect, which is an immune‐mediated regression of nonirradiated lesions following local RT [136, 137, 138]. A similar effect, in which RT would induce tumor cell death and expose tumor antigens, thereby priming the immune system for a broader response, has been proposed for RT in combination with immunotherapy, based on animal models and limited clinical data in solid tumors [139]. Whether bispecific antibodies could potentially boost the abscopal response by directing T cells to these exposed antigens throughout the body following local RT needs further study [140].

## 9 Conclusions

EMD is an aggressive form of MM, characterized by increasing independence of the tumor from the marrow microenvironment, resulting in the expansion of plasma cells outside the BM [3]. The mechanisms of EMD are complex, involving alterations in chemokine receptors and adhesion molecules, as well as genetic and epigenetic changes, such as mutations in the MAPK pathway, abnormalities in 1q, loss of *TP53*, and heterogeneous expression of the therapeutic targets BCMA and GPRC5D [7, 8, 12, 48, 51]. Increased heterogeneity of tumors and the microenvironment compared with MM in the BM has also been implicated in EMD [7, 38].

How EMD is defined has implications for the patient population included in studies and the prognosis associated with various therapies. Patients with bone‐associated PMD are generally older than those with bone‐independent, true EMD, and their outcomes with therapy are typically similar to those of the general MM population [13, 25, 107]. Patients with true EMD tend to be younger and have much poorer outcomes than those with PMD or no EMD [13, 25, 107]. Studies have not always analyzed these groups separately, which can be a challenge for interpreting data on epidemiology and treatment effects. Although PMD has historically been considered a form of EMD, there is growing consensus to distinguish patients with true EMD, as a distinct, particularly difficult‐to‐treat population [3, 14].

Imaging studies play an important role in the diagnosis and management of MM, and as modalities have evolved ^18^F‐FDG PET‐CT and whole‐body MRI with DWI have emerged as preferred methods of detecting and evaluating response in patients with EMD [14, 59, 62]. The combination of metabolic and morphologic assessment with PET‐CT, along with functional and morphologic assessment with MRI, allows for the identification of bone destruction with tumor burden and disease activity [60]. With its ability to quantitatively measure disease and to distinguish active and inactive tumor sites, PET‐CT has been used to identify predictors of outcomes based on the number of lesions and quantified FDG uptake [59, 60]. For patients with EMD, response assessment should always combine both laboratory and imaging evaluations to accurately monitor disease. Response criteria for soft tissue masses are only partially defined in the current IMWG guidelines; however, standardization is needed for images and results to be consistent across institutions [14, 141]. Emerging response criteria are shifting focus away from urine and serum markers and lesion diameter measurements and placing greater emphasis on functional imaging, particularly metabolic activity and MRI findings [141]. This reflects a more accurate assessment of disease activity, especially in EMD, and may allow more patients with EMD to meet inclusion criteria for clinical trials. Additionally, new tracers are being developed to increase sensitivity for diffuse BM plasma cell infiltration as well as the specificity for MM‐associated lesions [60].

Thus far, much of the data on outcomes of novel therapies in patients with EMD comes from retrospective studies or analysis of predefined subpopulations in broader clinical studies. Response rates in patients with EMD in the RRMM setting have typically been below 25% before the approval of immunotherapies, with short median survival compared to the RRMM population [11]. Novel immunotherapies (CAR‐T, bispecific antibodies, and ADCs) are associated with improved response rates in patients with RRMM who have EMD compared with those of patients in observational studies treated with other available standard therapies (e.g., chemotherapy‐based regimens) [80, 88, 107]. However, responses and duration of response tend to be lower in patients with true EMD compared with PMD or no EMD [107]. More promising outcomes have been reported from the RedirecTT‐1 Phase 2 study in patients with true EMD, employing a dual BCMA‐ and GPRC5D‐targeting approach with the bispecific antibodies teclistamab and talquetamab [121]. ORR was nearly 80% with durable responses over 1 year in most patients with EMD in this study, far exceeding the typical outcomes with standard therapies [121]. Due to the aggressive nature of this disease, off‐the‐shelf treatment options are critical for patients with RRMM who have EMD, to induce an immediate response and minimize the morbidity and mortality [107]. As therapies improve, more prospective and dedicated clinical studies will be needed specifically in the population with true EMD.

## Author Contributions

All authors (S.K., J.R., S.Z.U., Y.C.C., J.C.Y., M.‐V.M., V.H., and E.Z.) participated in drafting or revising the manuscript, and all approved the final version for submission.

## Funding

This work was supported by Johnson & Johnson.

## Ethics Statement

The authors have nothing to report.

## Consent

The authors have nothing to report.

## Conflicts of Interest

**Shaji Kumar** has received research support from AbbVie, Adaptive, Celgene, Johnson & Johnson, KITE, MedImmune/Astra Zeneca, and Takeda, and has been an advisory committee member for AbbVie, Adaptive, Celgene Johnson & Johnson, KITE, MedImmune/Astra Zeneca, Merck, Novartis, Oncopeptides, Roche, Sanofi, and Takeda. **Joshua Richter** has served in a consulting/advisory role for AbbVie, Bristol Myers Squibb, Genentech, Johnson & Johnson, Karyopharm, Pfizer, Regeneron, Sanofi, and Takeda, and has participated in speaker's bureaus for Adaptive Biotechnologies, Bristol Myers Squibb, Johnson & Johnson, and Sanofi. **Saad Z. Usmani** has served a consulting or advisory role for AbbVie, Amgen, Bristol Myers Squibb, Celgene, Genentech, Gilead Sciences, GSK, Janssen, Karyopharm Therapeutics, Merck, and Takeda, and received research funding from Amgen, Array BioPharma, Bristol Myers Squibb, Celgene, GSK, Janssen, Merck, Pharmacyclics, Sanofi, Seattle Genetics, and Skyline Diagnostics; **Yael C. Cohen** has served a consulting or advisory role for Amgen, Bristol Myers Squibb, GSK, Johnson & Johnson, Medison, Pfizer, Roche, Sanofi Aventis, and Takeda; has received research funding from Johnson & Johnson and Takeda; and participates in a speakers' bureau for GSK and Johnson & Johnson. **Jing Christine Ye** has served in a consulting/advisory role for and received honoraria from Bristol Myers Squibb and Johnson & Johnson and has received research funding from Celgene, Genmab, GSK, MingSight, Novartis, Pfizer, and Regeneron. **María‐Victoria Mateos** has received honoraria from AbbVie, Amgen, BMS/Celgene, GSK, Johnson & Johnson, Kite Pharma, Oncopeptides, Pfizer, Regeneron, Sanofi, and Stemline. **Vania Hungria** has received honoraria from AbbVie, Amgen, Bristol Myers Squibb, GSK, Johnson & Johnson, Pfizer, Regeneron, Sanofi, and Takeda. **Elena Zamagni** has served a consulting or advisory role for and received honoraria from Amgen, Bristol Myers Squibb, Johnson & Johnson, Menarini Stemline, Oncopeptides, and Pfizer.

## Data Availability

The authors have nothing to report.
